# Dr. J. R. Walker

**Published:** 1887-08

**Authors:** 


### Obituary.

---

DR. J. R. WALKER died at Bay Saint Louis, Mississippi, June 22, 1887. A tender and faithful husband, a kind and loving father, a sincere and true friend, he was devoted to his profession, which he loved next to his family. He had the courage of his convictions, and in his ardent nature maintained them with zeal, and often with enthusiasm. He was not perfect, as none of us are, but his mistakes may be attributed to this trait in his character, for he always pursued the course that he thought was right.

Let us extend our sympathy to his family, who have sustained the greatest loss, and keep green the memory of his many virtues.--*R. F. H., in Dental Cosmos.*

